# Pelvic organ prolapse and treatment decisions– developing an online preference-sensitive tool to support shared decisions

**DOI:** 10.1186/s12911-020-01264-1

**Published:** 2020-10-15

**Authors:** Mette Hulbaek, Eva Knutz, Niels Teglhus Ebbesen, Jette Primdahl, Jesper Bo Nielsen, Regner Birkelund

**Affiliations:** 1 Department of Gynecology & Obstetrics, Hospital of Southern Jutland, University Hospital of Southern Denmark, Kresten Philipsens Vej 15, 6200 Aabenraa, Denmark; 2 Department of Regional Health Research, University of Southern Denmark, Odense, Denmark; 3 Department of Design & Communication, University of Southern Denmark, Kolding, Denmark; 4 Department of Gynecology and Obstetrics, Odense University Hospital, Odense, Denmark; 5 Danish Hospital for Rheumatic Diseases, University Hospital of Southern Denmark, Soenderborg, Denmark; 6 Hospital of Southern Jutland, University Hospital of Southern Denmark, Aabenraa, Denmark; 7 Research Unit for General Practice, Department of Public Health, University of Southern Denmark, Odense, Denmark; 8 Lillebaelt Hospital, University Hospital of Southern Denmark, Vejle, Denmark

**Keywords:** Shared decision making, Preference-sensitive decisions, Pelvic organ prolapse, User involvement

## Abstract

**Background:**

Female patients with pelvic organ prolapse and clinicians need to take decisions regarding treatment that are often unpredictable in relation to how they impact the future everyday lives of the patients.

This study formed the developmental phase of a larger study to develop and test an online tool to support shared decision-making.

**Methods:**

Patients, health care professionals and other stakeholders participated in the development and evaluation process of this tool. The collected data was generated from observational studies, exploratory interviews with prompt cards and workshops with end users from four Danish gynecology outpatient clinics.

**Results:**

Content analysis led to important themes. For the patients three themes emerged: 1) how the impact of symptoms on everyday life affected the need for relief, 2) their bodily perception and sex life and 3) their worries about the future. For clinicians the different symptoms and their severity was a main theme.

**Conclusions:**

This article provides an overall description and discussion of the development methodology. It demonstrates how user involvement informed the prototyping process and how patients’ preferences were included in the final prototype. Whether the tool actually increases SDM, remains to be tested in a pilot feasibility study.

## Background

It can be complicated to elicit patients’ preferences during medical consultations in order to share decisions. This is because multiple factors arise during clinical decision making, such as interactions between the patient’s symptoms, uncertainty regarding treatment effect or the patient’s ability to understand the need for compliance with treatment. Health care decisions are rarely simple and are most often multi-criterial [1].

One example of a multi-criterial context is the setting of decision making for the condition of pelvic organ prolapse (POP) in gynecology. Here, patients have to make important choices between different treatments options. POP is defined as the lower abdominal organs, e.g., the bladder or uterus prolapsing into the vaginal vault [2]. The incidence of POP increases with age and, with 50% of women developing POP, it is a common finding [3]. POP symptoms often affect women’s quality of life considerably [4, 5]. Objective symptoms, such as urinary retention, severe constipation, urinary or fecal incontinence, occurrence of sexual problems and pain are seen, but also more subjective symptoms, e.g., perception of heaviness or dullness in the pelvic area are common [2, 6, 7]. Apart from surgery, other treatment options offer symptom relief to a certain degree and include pessaries, vaginal hormone treatment, pelvic floor exercises and dialogue regarding various lifestyle changes [2, 8, 9]. Lifestyle issues, e.g., obesity, inexpedient dietary habits, together with constipation, straining toilet habits and work involving heavy lifting can exacerbate symptoms. Some studies find evidence that occupational change to avoid heavy lifting, dietary changes, loss of body weight [2] and cognitive training on good voiding habits, or information about appropriate bowel habits can decrease symptoms [10].

The experience of specific symptoms can often be inconclusive for choice of treatment, because they are not correlated to severity or origin of the prolapse [11, 12]. Surgical procedures are effective for some symptoms, but can lead to new symptoms, e.g., dyspareunia or urinary incontinence [13]. Thus, clinicians need to know all important symptoms when diagnosing and offering a treatment plan for women with POP, but they also need to explore the impact of the symptoms on the patient’s daily life and explore the individual woman’s preferences and resources to comply with possible treatment options. In this multi-criterial context with multiple important criteria and unclear treatment effects, the overview for clinicians and patients and the decision making itself could become jeopardized. At the initial consultation, an exploration of symptoms as well as a clarification of patients’ preferences need to be correlated to available treatment options to offer good choices. Abhyankar and colleagues explored decision making in the context of POP with focus group interviews and found that women felt a lack of choice, of opportunity and support for involvement and a need for more patient-centered care [14]. To practice patient-centered care, shared decision-making (SDM) is a possible clinical practice to support patient involvement in the communication and decision making process [15].

### Current aids and tools for a process of shared decision-making

SDM is of growing interest all over the world in various health care systems [16]. According to a review performed by Makoul and colleagues, some of the most essential theoretical elements in SDM are 1) to explicate doctors’ knowledge, 2) to explicate patients’ values/preferences and 3) to present available options [17].

In the Danish health care system, SDM is not yet a standard practice among health care professionals despite good intentions [18]. A Danish survey from 2014 among 539 doctors and 824 nurses, found that the clinicians want to involve patients in their practice but think implementation is difficult due to lack of resources, knowledge and methods [19]. Elwyn and colleagues developed a model for SDM in the clinical practice [20]. The model presents a method with three important domains, which they suggest applied iteratively in the communication process and refer to as ‘talks’ (ibid.):

- The team talk
- The option talk and
- The decision talk

During an ‘option talk’, alternative treatments choices are discussed and, finally, a ‘decision talk’ leads to preferences being elicited and eventually to informed and preference-based decisions (ibid.).

In continuation of Elwyn’s model, Stacey and colleagues have looked at the role of patient decision aids (PDAs) [21]. PDAs are tools whose aim is to support patients’ involvement in decision-making; they are useful in the option talk and decision talk in particular. PDAs should apply to a set of international standards – e.g., the International Patient Decision Aids Standard (IPDAS) [22–24]. PDAs include methods/strategies to help patients clarify their values in relation to options and to integrate these into the decision making process. PDAs should provide sufficient information for the decision making process. Nevertheless, it can be complicated for patients to grasp the amount and complexity of necessary information e.g., information on health condition, on all options, harms, disadvantages, side effects, outcomes and probabilities. Often, patients do not believe in their own ability to understand all the information given in consultations [25, 26].

Thus, to support SDM in this complex multi-criterial setting a new tool could aim to combine symptoms, their interactions with evidence-based data or best estimates of outcomes, and correlate this with patients’ subjective perspectives. Multi-criteria decision analysis (MCDA) is a technique, that based on an algorithm, presents the best option according to the patients’ preferences [1, 27]. The assessed presentation (a ranking of options) is calculated according to the extent to which an option creates value through meeting a set of criteria (ibid.). One example could be the MCDA aid to help with contraceptive choices [28] or the internet-based PDA for prostate cancer screening [29]. Existing PDAs for use in the context of gynecological patients with POP are developed in accordance with the international IPDAS criteria but they lack integration of the individually elicited preferences into generated value-based options that the MCDA technique offers [30, 31].

In this project, the scope was to develop an IT-based tool with an MCDA algorithm to support patient involvement through the concept of SDM, by means of including revealed preferences from women suffering from POP. The tool is meant to become a steppingstone for the subsequent communication process. Many of the elements from ordinary PDAs e.g., additional information about all relevant treatment options and their pros and cons, will be introduced by the clinician during the consultation subsequent to the discussion of the MCDA presentation. In the tool, an integrated patient survey elicits patients’ preferences. This elicitation clarifies the individual woman’s values related to a range of user chosen criteria from an analysis of field data, e.g., costs of or possible risks associated to the available options. The algorithmic functionality within the tool will combine patients’ preferences with prefixed evidence-based data or best estimates from clinicians, in relation to each different option. The tool presents the different options in ranked order in a graphic presentation within the patient’s online medical journal. The ranked options should kick-start a communication process to support SDM especially the option talk and the decision talk during the consultations. A development phase sets out to develop the IT based tool with the MCDA algorithmic functionality. Subsequently a test phase will follow to test the feasibility of the tool in the real world.

This paper describes the development phase of the larger study: Development and testing of an online tool for patients with POP to support SDM. Thus, the aim of this paper is to provide an overall description and discussion of the first development phase. The paper describes the development methodology and describes how results from field research have informed the prototyping of the tool.

## Methods

### Overall description of our methodology

Our approach involved stakeholders within decision-making regarding patients with POP. Stakeholders, besides the patients, were physicians, nurses and physiotherapists from gynecological outpatient clinics where the women had outpatient medical consultations, and organizational managers from the departments that were responsible for organizing health care for these patients.

To explore the context of decision-making during the consultations, the methodology of contextual design [32] was chosen as an overall approach. This user-centered approach foregrounds “the context” when framing the design process. Specific to contextual design is the contextual inquiry. The inquiries aim to explore and understand the users by combining collected data from participant observations with interviews to interpret the collected data (ibid.). This should be done whenever possible through the development process.

According to the contextual design model, the development consists of: 1) gathering user data, 2) designing (drafting /prototyping) and 3) testing [32]. Thus, our development method consisted of the following three stages:

- Stage 1: Field research – to gather data to achieve an understanding of user needs in the context of gynecological medical consultations through field observations, interviews and workshops.
- Stage 2: Designing – through an iterative process of drafting, testing and re-drafting.
- Stage 3: Testing – through the iterative process of testing and re-testing the newly emerged drafts, leading to a final prototype for the subsequent feasibility test.

**Fig. 1:**
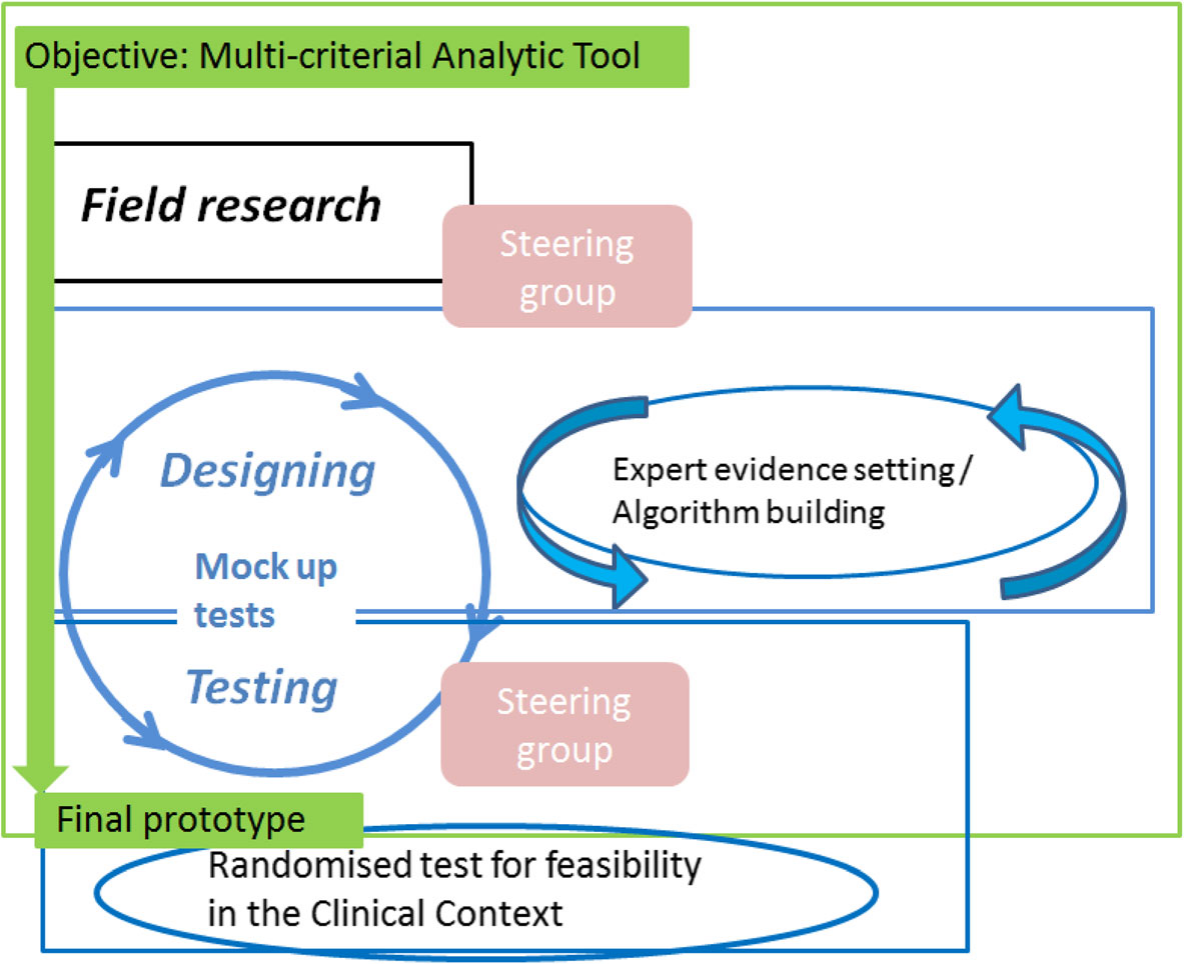
Overall method

Figure 1 illustrates the overall method in accordance with the contextual design model [32]. Important to notice is the combination of the design and test stages in an iterative process, with the aim of gleaning new knowledge about and understanding of user needs and to enable new ideas brought up by the users to be applied in changing the draft. Ideas were tested on the spot (re-drafting and re-testing). Tests in the development phase involved electronic screen-based mock-ups (see Fig. 3). These allowed tests for comprehensibility on single elements, as well as tests on usability of the overall tool including the patient survey, the MCDA algorithm and the interface within the patient’s medical journal. Finally, a feasibility test was planned to evaluate the perceived effect on the SDM process and the usability in a clinical setting with real life decisions. The feasibility test is not part of this paper.

#### Project groups

Besides the research team (the authors MH, NE, JP, JBN and RB), the project involved two important project groups: the steering group and the MCDA group. The steering group involved key stakeholder representatives and was established according to IPDAS [23] to follow the development throughout the three stages. The steering group included: 1) three patients with POP who had been through decision making themselves, 2) six clinicians (gynecologists, continence nurses, physiotherapists) and 3) two organizational managers from departments where the tool would be implemented. The purpose of establishing the steering group was to democratize the development process and to ensure that different voices had been heard. In this regard, the steering group had to agree to the final prototype and lead the direction of the tool during the early development phase of field research (stage 1) and, later, to approve of the final prototype after the design and testing stages (stages 2 and 3).

Besides the steering group, the research group appointed an MCDA group. This group consisted of three consultants involved in daily practice, one professor in the field of gynecology and one professor in the field of decision-making and risk communication. This group conducted several workshops through stages 2 and 3 to establish evidence and best estimates for the MCDA algorithm within the tool. Statisticians advised the group on how to build the algorithm within the tool where evidence or best estimates should be combined with the patients’ preferences in relation to different treatment options.

Further, five IT specialists with technical development competencies were associated with the project and one independent communication specialist, to offer text literacy and communication competencies for the survey text.

#### Project participants

The development of the tool was performed from September 2016 till March 2018 by involving four different Danish outpatient clinics. To gain diversity of perspectives, we involved patients with different symptoms from their prolapse, different treatment decisions, different educational and social backgrounds and different ages. The age span of the entire group of patients participating in interviews, expert groups and tests ranged from 30 to 80 years of age.

A total of 62 individuals played an active role in the development: 25 patients, 26 clinicians, seven secretaries and five organizational managers. The tests included 57 test scenarios. Ten workshops were conducted that included patients, clinicians, managers and statisticians.

### Details in relation to field research (stage 1)

**Fig. 2:**
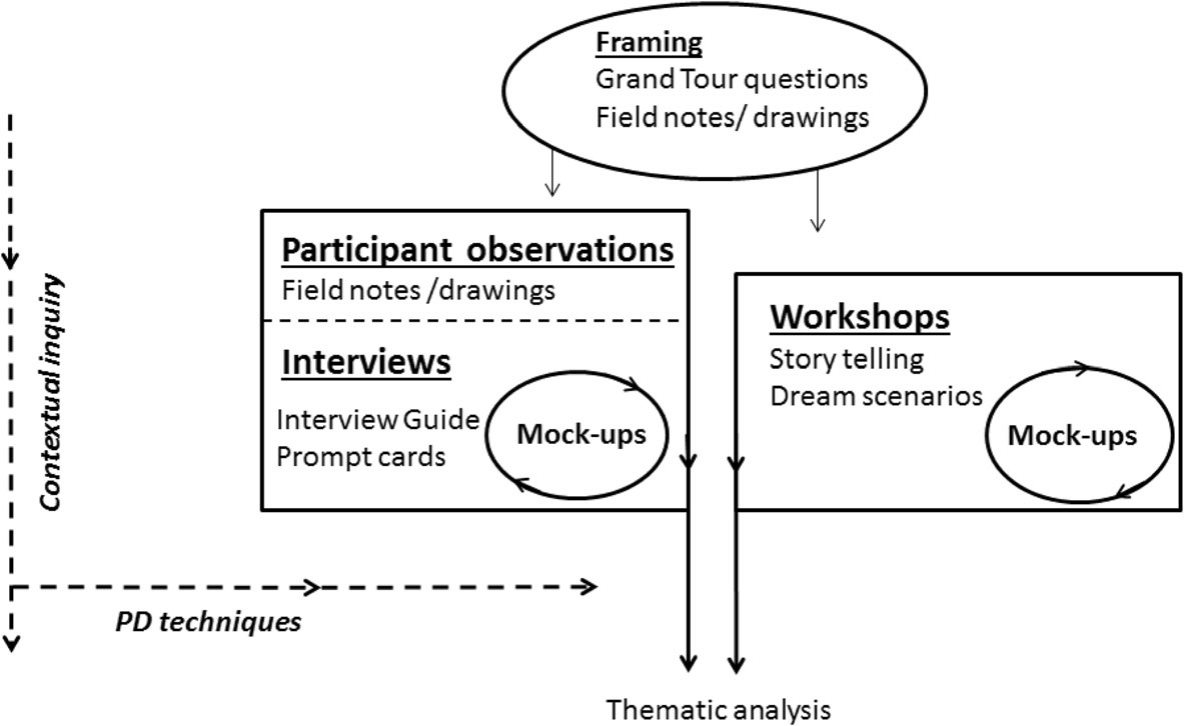
Field research

This section will detail the methods used during the field research (stage 1) that took a particular user-centered approach. Figure 2 illustrates the methodological approach of stage 1.

**Fig. 3:**
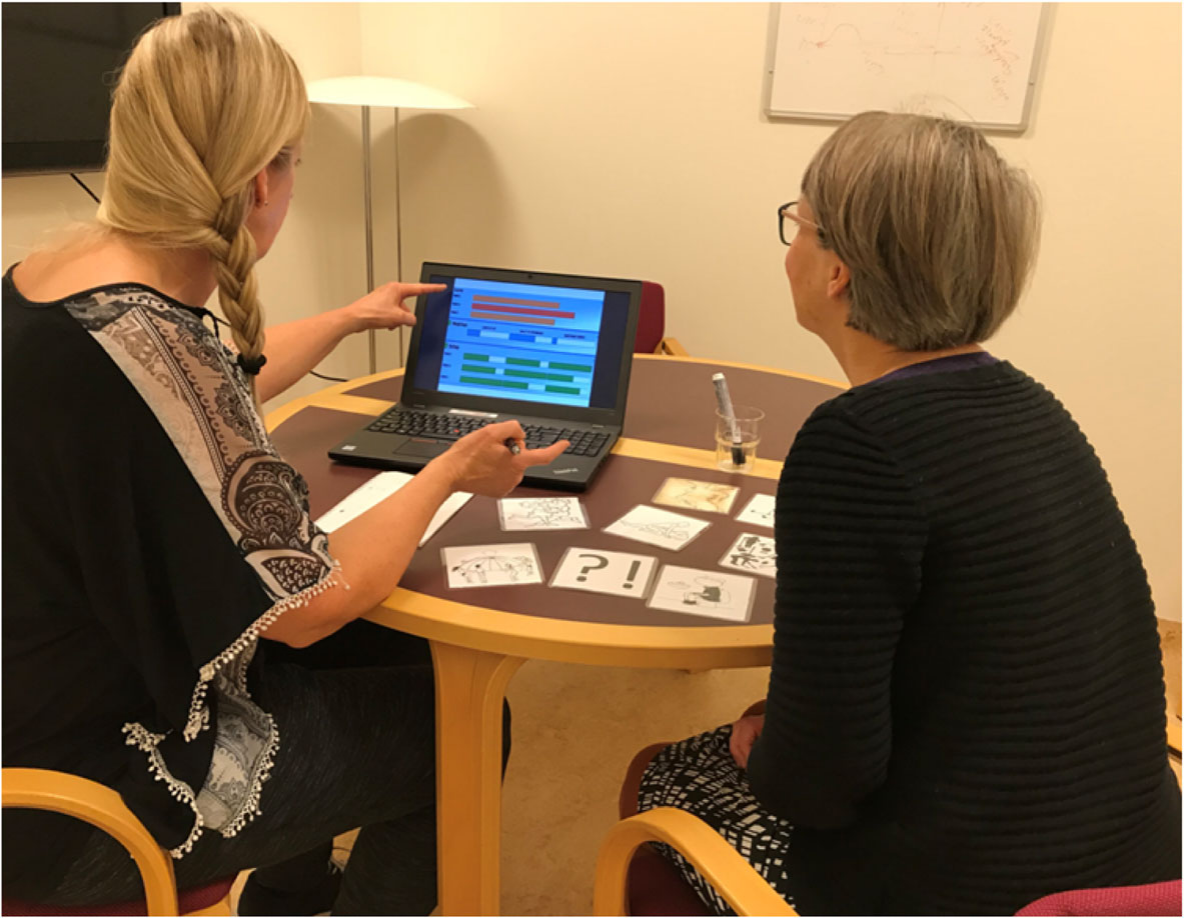
Staged situation from an interview showing the use of mock-up and prompt cards

**Fig. 4:**
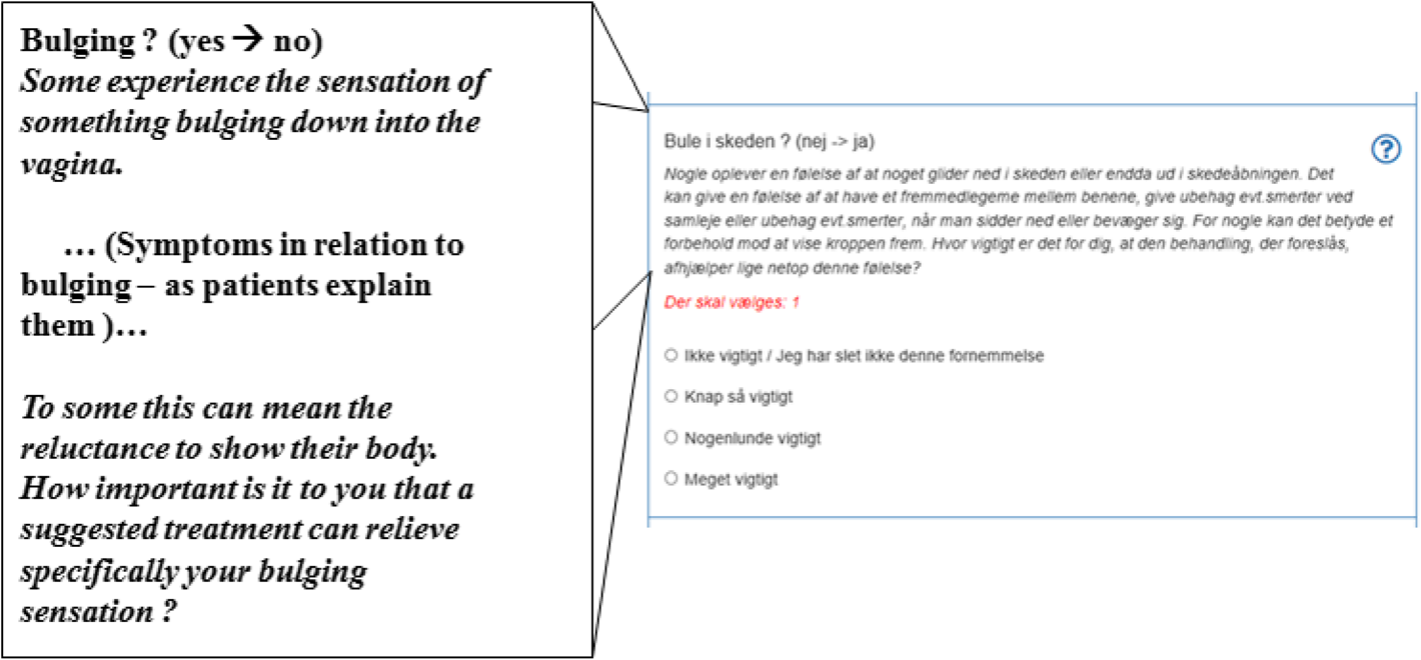
Screenshot - Survey question (Danish with English translation) about bulging symptoms

**Fig. 5:**
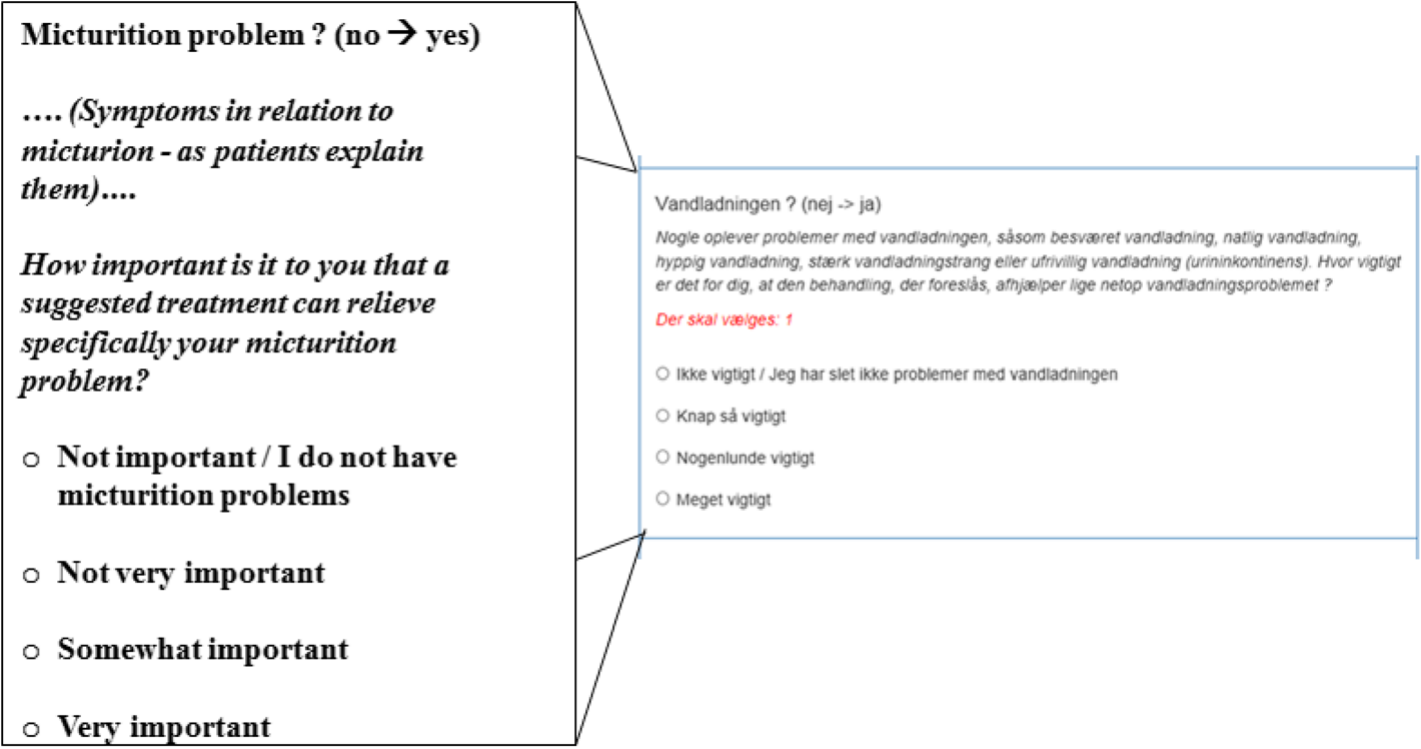
Screenshot - Survey question (Danish with English translation) about micturion problems

In planning the overall framing of the development phase, field observations at the outpatient clinics were conducted, with inspiration from Spradley’s grand tour questions [33]. Field notes and drawings from the preliminary field observations helped frame: 1) the participant observations during the consultations, 2) the development of an interview guide for the interviews and 3) the manuscript for the workshops.

Through interviews and workshops, patients and clinicians participated in identifying themes that generated the important criteria regarding decision making during consultations. The aim was for the criteria to provide preference-eliciting questions for the survey. The field research was conducted by an insider researcher, the author MH, who is a continence nurse with 19 years of experience in the field of gynecology. This experience could strengthen the study because the researcher would be able to question and explore important information during the inquiries given her specific prior knowledge of POP and the treatment traditions and options in Danish gynecological health care setting. On the other hand, it could limit open-mindedness as a prerequisite to the research methodology [33]. To preserve open-mindedness and to achieve valid data and results during the development phase, we involved: 1) the steering group with patients and clinicians that gave input to the findings and their interpretation, 2) the expert group to discuss the construction of the MCDA functionality, and 3) co-researchers in data collection during workshops. Finally, during the whole development phase, the experts on qualitative research from the research group discussed findings and interpretations.

Besides qualitative research methods and contextual inquiry, stage 1 involved techniques from participatory design [34–36] such as the use of prompt cards during interviews and dream scenarios and storytelling techniques during workshops. Prompt cards are pictures that can prompt and capture inspiring or emotional experiences [35]; in this way, prompt cards prompted for stories from the participants. These techniques enabled patients and clinicians to express and generate design proposals for the subsequent professional IT development work. Procedures concerning participants observations, interviews and workshops are specified below.

#### Participant observations

Eight consultations at three hospital departments were observed to collect the individualized patient data for the following interview and inquiry.

During participant observations, the observer took field notes focusing on the three talks from the model of Elwyn and colleagues; focusing particularly upon the option talk and the decision talk. Further, the measure of the overall trend of SDM in the consultation was to be discussed with the patient. The observer used a measuring tool, called the OPTION^5^ Observer TM [37, 38], to evaluate this trend. Here a number of areas within SDM has to be evaluated, generating an overall score ranging from 0 to 20 points. A high score indicates a high degree of observer-perceived SDM.

#### Interviews

Ten interviews with patients aged between 41 and 80 years were conducted at the three hospital outpatient clinics. They lasted between 18 and 56 min (a mean of 33 min). Eight interviews took place immediately after observation of the patients’ consultations. The interviews aimed to identify themes and criteria in the decision making from the patients’ perspective and to reveal other important needs for the tool.

The field notes from observations were used during the individual interview to explore the patient’s needs and together with the patient to interpret the contextual influence of the setting in relation to her decision-making.

Eight prompt cards were used during the interviews placed in random order. One of the cards envisioned a question mark like a kind of a blank card where women could think of their own criteria/factors important to them. The remaining seven cards envisioned pictures from specific areas, which, from the evidence of qualitative research, seemed to be important for women with POP [39, 40]. They illustrated: 1) a working situation, 2) the female body, 3) feeling of pain, discomfort, 4) leisure activity, exercise with others, 5) a dancing situation, 6) sex life, and 7) toilet visits. During the interviews, we introduced an online screen-based freely available mock-up as an example to visualize and discuss the concept of MCDA [41]. During the mock-up, the patient was encouraged to discuss her own ideas for criteria that aligned with her own personal preferences (Fig. 3).

The use of prompt cards and the online screen-based mock-up were optional for the patient in the interview situation. Eight patients used mock ups and four patients picked prompt cards during interview. None of the participants picked the card with the question mark.

All interviews were audio-recorded and thematically analyzed according to Steinar Kvale’s methodology of coding, condensing and categorizing [42] through the conceptualization of the three-talk model for practice of SDM.

The audio-recordings were listened through once to achieve a sense of the whole and afterwards listened through several times sentence-by-sentence with a focus to identify interesting or important text sequences (meaning units of phrases, sentences and sections) which led to the initial coding. The coded text was condensed if necessary, and finally grouped into themes that included the criteria for the survey questions.

#### Workshops

In total, five workshops lasting between 1½ hours and 3 h (a mean of 2 h) were conducted with the participation of 21 clinicians (gynecologists, continence nurses and one physiotherapist) and five patients from four Danish hospitals. The workshops aimed to identify themes and criteria for decision-making primarily from the perspectives of the clinicians and to reveal other important needs and treatment options for the tool. The first author, MH, acted as moderator and facilitator, together with the clinicians, and explored different angles on the clinicians’ self-experienced stories. We used storytelling to engage clinicians actively in co-designing. Three stories that were collected during field observations were used to prompt new stories about: 1) successful and unsuccessful decision-making situations, 2) good and bad rapport situations with a patient, and 3) a challenging consultation regarding decision-making. Next, the clinicians were encouraged to create a story about an imaginary ideal tool for an SDM situation (a dream scenario) followed by an analysis of: 1) strengths and weaknesses of and 2) pros and cons of their “imaginary” tool. For this, they used a framework called SWOT analysis – analyzing Strengths, Weaknesses, Opportunities and Threats [43].

We introduced the same online screen-based mock-up that was previously used during interviews and this mock-up draft was further elaborated and discussed in workshops.

All the generated data from the workshops were written progressively on sticky notes by the different participants. Afterwards, the moderator (MH) read the sticky notes several times to identify interesting or important text sequences (meaning units of words, phrases, sentences and sections). These inputs were then coded, condensed and thematically organized, according to Steinar Kvale’s methodology [42].

## Results from field research

### Field notes for inspiration during framing

During the field observations, we used field notations, drawings and grand tour questions to gain information about the contextual setting of the consultation. This information helped frame the subsequent interviews and workshops. From observations, we learned that there was a need among the clinicians for support to clarify patients’ preferences if they were to practice SDM in the multi-criterial setting. It seemed to be a challenge to have the necessary time for reflection for both clinicians and patients.

After a consultation, a consulting gynecologist described the challenge of a consultation:

*You send along several different arrows towards the patient in your conversation. Often you have to work your way to the important inner circle after several attempts in different directions. You hope to be skillful enough to hit the target in time.*

A nurse specialist expressed that elderly patients often were overwhelmed and taken by surprise by how fast things work from the initial consultation to actually getting surgery. In her view, patients often lacked the time for reflection and she knew for a fact that sometimes patients after their decision-making consultations had doubts about whether to go on with planned surgery.

The measure from the OPTION^5^ ObserverTM showed a mean of six points (range of 1–11) from the observed consultations. To understand the presented information and risks, the deliberation of options and pros and cons, and the presentation of all reasonable options, together with the support to compare alternatives were the areas that scored low and thereby expressed a trend of challenges for SDM.

### Findings based on prompt cards from interviews

Four patients used the prompt cards during the interviews. In Table 1, an overview of some of the quotes prompted by prompt cards and the analysis is provided. Through the analysis of the interview data, three overall themes emerged:

- How the impact of symptoms on everyday life affects the need for relief
- Bodily perception and sex life
- Worries about the future

**Table 1:**
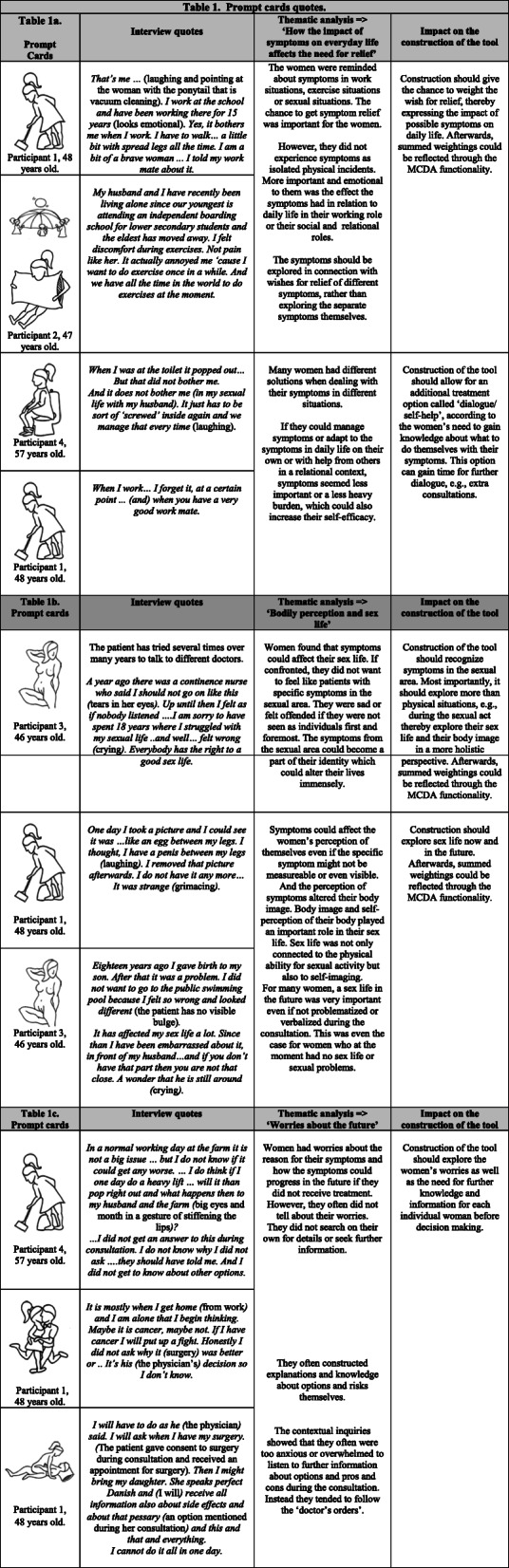
Prompt cards quotes

Table 1 also shows how the themes led to conclusions that impacted the construction of our tool.

### Findings based on workshops

One workshop where clinicians participated together with patients revealed no additional findings compared to the data generated during the individual interviews with patients.

However, the thematic analysis from the remaining four workshops without patients revealed one main theme and one subtheme, which were specific for clinicians:

- The different symptoms and their severity (main theme)
- Knowledge about the patient in relation to options (subtheme)

The workshops revealed that clinicians would like a tool to work systematically through symptoms according to manuals. Clinicians focused primarily upon how to explore symptoms and interpret them from a diagnostic perspective.

In the literature, Abrams and colleagues [2] describe four groups for standardization: 1) symptoms from the micturition function, 2) symptoms from the bowel function, 3) symptoms of bulging or pressure and 4) symptoms regarding the sexual function. In the dream scenario, clinicians imagined the tool to be a checklist for the national guideline and this common system of standardization. Thus, analysis of the workshop data revealed that according to clinicians’ perspectives, the ability to define symptoms assured the best clinical decisions and, the best option talk, leading to the best decision talk. They felt that the absence or presence of these symptoms would be the primary clue to good clinical decision making. Subsequently, they would explore the patient’s perspective. A consulting gynecologist expressed at workshop 5:

*It is also good to get to know what is in her ‘rucksack (of symptoms)’, so to speak, … in that way we can be more sure not to miss out upon explaining important stuff and not to misunderstand each other (in relation to finding what options to offer).*

In this way, the clinicians needed to ensure that all symptoms from the guidelines were systematically explored, and this affected the construction of the tool.

### How did the prompt cards inform the prototype?

In relation to the two themes ‘How the impact of symptoms on everyday life affects the need for relief’ and ‘Bodily perception and sex life’, prompt cards and stories revealed that the women – as opposed to the clinicians – did not only see symptom relief in the common light of variation and intensity, but also in relation to more individually important facets of their lives.

The prompt cards revealed that survey questions should be able to differentiate between physical measureable symptoms, i.e. the degrees of descent or visibility of a bulge and the patients’ psychological perception of bulging. Figure 4 shows how a survey question opens the possibility to express affected bodily perception regarding the phenomena of bulging. The MCDA functionality and its algorithms then give the possibility that elicited preferences affect each option.

In relation to the theme ‘Worries about the future’, prompt cards also revealed that the women speculated about the origin of their problems and about future aspects of their symptoms (Table 1c). The theme showed the women’s need for further dialogue and discussion with additional information being offered, e.g., additional consultations to allow for reflection. This need for reflection could be compared to the need of ‘no treatment’ but was encompassed in the MCDA functionality as a specific option with an algorithm of its own to create awareness of the patients’ need for reflection. This option was presented graphically, and was called ‘dialogue/ self-help’ and contained 1) further exploration of the patients’ preferences and 2) basic guidance upon life style changes, cognitive training or information regarding pelvic floor exercises, and 3) the possibility of additional follow up visit(s).

### Criteria and weightings

By analyzing all the findings from interviews and workshops, we found 16 important criteria. The criteria were grouped into three domains of questions that finally encompassed all criteria: 1) a domain for symptoms, 2) a domain for willingness to take certain risks regarding treatment, and 3) a domain for willingness to and belief in own ability of investing resources in the treatment (Table 2).

**Table 2:** Criteria for the domains of questions

Important criteria -Derived from the five themes found in field research	Domains	Impact on questions
Hormone level	**The domain of symptoms**	**Led to 1 question about hormone level**
A feeling of heaviness		**Led to 7 questions in the survey** Introductory text: What problems do you want the treatment to address? E.g., question 3 (bulging) (Fig. 4) or question 4 (micturion) (Fig. 5).
A bulge in the vagina		
Micturion problems		
Defecation problems		
The impact of the problems on sex life		
The importance of sex life in the future		
Worries and thoughts		
Willingness to take risks in relation to sudden treatment-related problems	**The domain of risk**	**Led to 3 questions in the survey** Introductory text: Risk taking / Your willingness to take risks
Willingness to take risks in relation to transient treatment-related problems		
Willingness to take risks in relation to long-lasting treatment-related problems		
Hormones - applied in the vagina	**The domain of effort**	**Led to 5 questions in the survey** Introductory text: Your willingness and ability for certain treatment consequences
Sick leave		
Financial expenses		
Pelvic floor muscle training		
Lifestyle changes		

The patient weights each criterion when she answers a correlating question in the survey. Each possible answer would have a correlating proportion to each treatment options’ algorithm, and by choosing a specific answer to the specific question, the patient expresses her preference, which equates her weighing of the importance of the specific criterion. An example of weighting through questions can be seen in Fig. 5.

Findings from field notes, interviews and workshops also indicated that sufficient time for reflection should be given for patients to clarify their preferences. Therefore, the survey preferably should be available before the consultation (home-based) and with adequate time-out to answer questions in the system.

## Discussion

In this section, we discuss the applied methodology in relation to user involvement and participation and how this particular approach informed the development of our final prototype.

### User involvement in research

In the development of PDAs, a core element is the involvement of the users [23, 24]. Involving users seems to be of positive value for system success and user satisfaction [44]. Reasons to involve users in the development of PDAs using health IT could be: 1) to increase functionality and quality, and 2) to give voice to those who are impacted by the IT function [45].

In our project, we accomplished giving voice to the users. We added techniques from the participatory design method to give voice to and further motivate the users by giving them their say in the development process [36]. Participatory design is used increasingly in health care research when developing IT support [46] and PDAs [47]. We established a steering group with patients and clinicians from within decision-making, but we also gave voice to additional stakeholders, i.e. 1) nurses and physiotherapists that could be essential to the patient in their reflection during the decision-making process, and 2) organizational managers from the outpatient clinics that would be important for optimal future implementation of the tool. Furthermore, we gave a say to patients through the steering group and thematically analyzed interviews that, compared to common guidelines, created alternative themes and options that represented patients’ perspectives. Through the design with contextual inquiries, patients’ ideas created new drafting and retesting, which finally evolved into a prototype for our online tool to elicit women’s preferences for treatment decisions.

Involving patients in health care is on the political agenda in many countries today. In Denmark, patient-centered care has been put on the national agenda [18]. In the Danish health care system, patients and their relatives should be involved in treatment and health care, and this is monitored in relation to national quality targets [48]. The involvement of patients in health care research as partners is increasing [49] and can be imperative in being awarded research funding. The assumption is that the quality of the research increases if patients who are potential recipients of the health care services are involved in all aspects of research (ibid). Standardized processes, e.g., IPDAS [23, 24] and the mapping of the development process [50] should be applied when developing PDAs, in order to ensure that a certain level of quality in methodology is achieved. However, in the current study, the assessment of needs for the decision making process called for a preference-sensitive tool to get the communication process ‘kick started’ towards informed decisions and SDM and for a tool which could be easily applied through the medical record to be used in consultations. Since it was not a PDA in its original sense, we could not adhere to the common quality criteria for PDAs e.g., providing information about options in sufficient detail for making a specific decision. However, we resembled the methodological framework from the IPDAS Collaboration [23] by 1) scoping and assembling a steering group, 2) assessing patients’ and clinicians’ views, 3) determining a format and planning distribution, 4) collecting evidence, 5) drafting a prototype, 6) testing usability and 7) testing for feasibility (a planned pilot study protocol). We succeeded to strengthen the usability of the tool by involving patients in the development by applying participatory design techniques and the contextual design method where context and iterative drafting and testing is a core element of the development process. The development of the tool gives us the possibility to extend the tool later if needed.

### Prompt cards as an important part of user involvement?

The use of prompt cards was an essential element in the construction of our tool. Like cultural probes [51, 52] prompt cards can lead to inspiring responses by evoking good and bad memories from the past, and hopes and dreams for the future (ibid.). However, this method can induce uncertainty if uncritically used in a scientific process [53]. One could argue that our data collected through prompt cards would not be comprehensive and adequate to capture all facets of living with a pelvic organ prolapse. Our ambition, however, was merely to inspire the development of the tool by combining this inspirational data with more traditional ethnographic data from e.g. thematic analyzed interviews.

Prompt cards showed that for the patients the perception of a symptom was depending upon interaction with other symptoms or with how it affected their role in everyday life. Clinicians, on the other hand, wanted to explore symptoms with a diagnostic angle and as separate and categorical incidents following standardizations and common questionnaires [54, 55]. We believe that if we developed our tool as according to ordinary guidelines and checklists, the tool might call for what medical sociologist Arthur Frank calls instructed stories about the patients’ symptoms rather than their individualized stories [56]. Furthermore, according to contextual design, Holzblatt and colleague argue that users of IT have to live through extraordinary events, which reflect real life experiences and not abstractions in order to increase usability. We need to acknowledge that illness not only talks to a person’s brain but is also a bodily experience [56]. The different prompted stories contained feelings, which showed us different bodily experienced angles that were important to capture. This inspired us when creating survey questions and we believe that this method brought patients’ perspectives into the tool.

### Methodological reflections

Collecting and analyzing data from observations, interviews and workshops requires qualitative research skills and reflects interpretation. The methodology employed, the findings and the analyses were discussed with both the research team and the steering group during the development phase to ensure quality. A broad variety of stakeholders were identified and participated in the development, which is considered a strength. Furthermore, patients were recruited from several different outpatient clinics with various symptoms, ages and occupational backgrounds. Clinicians were both male and female and with different educational backgrounds.

The chosen methodology is very time consuming. The time taken to collect the great amount of data, through field research, interviews and workshops, steering group meetings, iterative testing scenarios for iterative drafting before a final prototype emerged, not only helped ensure quality, but also required an enormous effort from researchers, patients and clinicians and vigorous project management competences.

The development of a new functionality in the form of an online tool in an already existing online system required effective collaboration with skilled IT consultants to overcome unanticipated obstacles, e.g., fitting the new requirements from users with the existing and often inadequate software. However, this adaptation to an existing system had the advantage that it was less time consuming and less costly compared to developing a new support system for our tool [52]. In addition, it will make it more likely to be used in routine practice and facilitate implementation.

Whether the invested effort equals the benefit from the tool to patients and clinicians has yet to be determined in the final feasibility test.

## Conclusion and perspectives

In this article, we have given an overall description of our development methodology and demonstrated how empirical data from observations, interviews and workshops involving patients, health professionals and other stakeholders, has informed the development of a tool to support the shared decision-making process in relation to patients with POP. We have demonstrated how user involvement and design methods ensured that the patient’s preferences were taken into account. We set out to develop a tool for more preference-sensitive decisions with the use of MCDA. The prototype of the tool can support the communication process between caregivers and patients in clinical practice by potentially assisting them in a more preference-sensitive dialogue. The tool has some limitations if it is to be compared with ordinary PDAs developed in accordance with the IPDAS criteria. However, we hope that our tool can help a shift towards increased SDM in consultations. This remains to be tested in a randomized feasibility pilot study.

It is fair to assume that the experiences of the applied methodology and the final structure of the prototype can be transferred to other health care areas. Furthermore, the findings from our field research can be used when planning future healthcare services to patients with POP.

## Data Availability

The datasets used and/or analyzed during the current study are available from the corresponding author on reasonable request.

## Abbreviations

PDA: Patient decision aid
POP: Pelvic organ prolapse
SDM: Shared decision making
IPDAS: International Patient Decision Aids Standard
MCDA: Multi-criteria decision analysis
